# Heavy Metal Concentrations in Fish from River Tano in Ghana and the Health Risks Posed to Consumers

**DOI:** 10.1155/2021/5834720

**Published:** 2021-11-08

**Authors:** A. J. Nyantakyi, S. Wiafe, O. Akoto, Bernard Fei-Baffoe

**Affiliations:** ^1^Environmental Protection Agency, P. O. Box GS 166, Ahafo Regional Office, Accra, Ghana; ^2^Sunyani Technical University, Sunyani, Ghana; ^3^Department of Chemistry, Kwame Nkrumah University of Science and Technology, Kumasi, Ghana; ^4^Department of Environmental Science, Kwame Nkrumah University of Science and Technology, Kumasi, Ghana

## Abstract

Appreciable levels of trace metals have been reported in the Tano basin, but data on the corresponding levels in fish and the risk they pose to consumers are limited. The levels of 7 trace metals in 18 fish muscles were assessed between November 2016 and October 2017 using acid digestion and PerkinElmer (PinAACle 900T) Atomic Absorption Spectrophotometry. Apart from Cu, all the metals studied were detected in all fish samples. The levels of Cr, As, and Hg were higher than the acceptable levels of fish muscles. Cr concentration ranged from 16.10 ± 0.2 mg/kg in *Clarias gariepinus* to 57.9 ± 4.2 mg/kg in *Sarotherodon galilaeus*. The levels of As ranged from 1.01 ± 0.08 in *Clarias gariepinus* to 3.00 ± 0.01 mg/kg in *Mormyrus rume*. Hg level was 0.58 ± 0.69 mg/kg in *Oreochromis niloticus* and 2.52 ± 0.70 mg/kg in *Ctenopoma kingsleyae*. However, Pb, Zn, and Cd concentrations were below the Food and Agriculture Organization limits with low target hazard quotients in all fish samples, suggesting no possible noncarcinogenic risks to adult consumers. Possible noncarcinogenic and carcinogenic health risks were recorded for As, Hg, and Cr in all fish species. Strong associations were observed between Hg, As, Zn, and Cr and between Pb and Cd suggesting a possible common source. *Mormyrus rume* fish species was under stress in the river, but the remaining species were in good condition. Periodic monitoring of trace metal concentrations in fish and enforcement of the buffer zone policy are recommended.

## 1. Introduction

Fish continue to be the main source of protein worldwide, yet trace metal pollution endangers them [1, 2]. Fish protein has some nutritional and therapeutic benefits [3]. Trace metal pollution originates from natural and human activities, including industrial effluent discharges, atmospheric deposition, mining, agricultural runoffs, and urbanization [4, 5]. Runoffs from agricultural activities contain appreciable concentrations of trace metals [6]. Fish in heavy metal polluted water are susceptible and vulnerable to toxicological problems because of their feeding habits and location in water environments [7–9]. Fish exposure to toxicants has had some harmful effects on their quality, diversity, and health of humans who depend on them for their protein needs [10]. For example, higher concentrations of Cd above 0.5 mg/kg in fish alter carbohydrate and protein metabolism [11]. Trace metals in sediments and water threaten aquatic life [3, 9]. This is due to their bioaccumulation, biomagnification through the food chain, and their potential threats to human health [12,13]. Metals deposited in rivers are immobilized and deposited into sediment through adsorption onto suspended particles, ion exchange with organic matter, incorporation into the mineral lattice structure, and precipitation as insoluble metals [12, 13]. In the water column, trace metals presence is enhanced by their solubility, mobility, and adsorption properties in the medium [14–16]. Metals including Cu and Zn in smaller concentrations are said to be important for the regular physiological and central functions of organisms including fish [17]. However, higher concentrations result in toxicological problems [18].

Fish concentrate heavy metals in their tissues and for this reason may be used in estimating the level of pollution in the aquatic ecosystems [19, 20]. The concentrations of micropollutants in fish depend on the body size, age, location in the water, physicochemical properties of the water, and their feeding habits [21]. The consumption of fish with heavy metals above the recommended limits leads to health problems including kidney, liver, brain, nervous, and skin complications and death [10, 17, 22]. Consequently, increasing metal concentrations in fish has been a grave global concern over the decades [23, 24].

In fast moving rivers, fish can travel through greater distances [2]. However, their movements may be impeded by physical obstacles, changes in pH, temperature, and turbidity. Studies conducted by Nyantakyi et al. [25] on trace metals in water and sediment samples from the Tano Basin have revealed appreciable levels of Hg, Cd, and As. Fish exposed to higher contaminants, including heavy metals, absorb the bioavailable forms directly from the aquatic environment [12, 26]. Communities located in the downstream of River Tano depend on fish from the river for their protein needs. However, data on the concentrations of trace metals in fish from River Tano and the risk they pose to the consumers are limited. This research sought to assess trace metals levels in the muscles of fish samples from River Tano in Ghana and the hazards they pose to consumers. The information could also be used to adopt some pollution control strategies and make informed decisions.

## 2. Materials and Methods

### 2.1. Study Area

**Figure 1:**
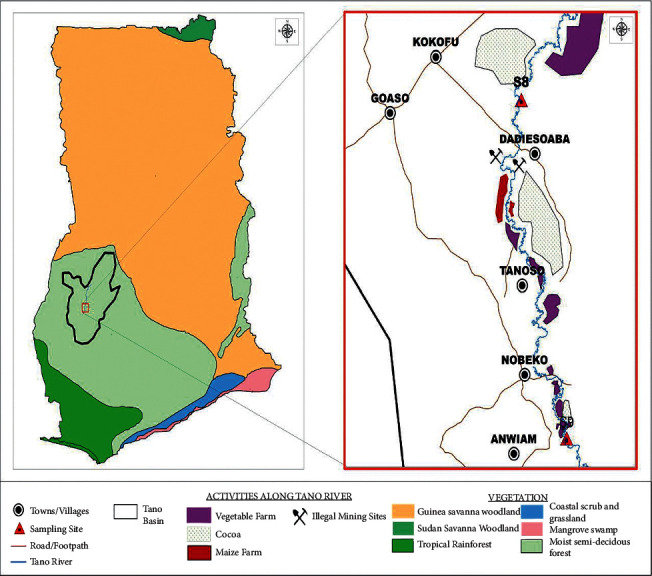
Map of Ghana showing the Tano River catchment in the Asunafo South District, sampling sites, and the land use.

The midstream end of the Tano Basin, which covers the Asunafo South district of the Ahafo Region of the Republic of Ghana, was used in this study. Communities within the area are into commercial and subsistence farming, mainly maize, cocoa, and vegetable farming (Figure 1). There are also pockets of fish farming and illegal mining activities in the study area. The area is relatively flat with moist semideciduous land cover [1, 27]. The vegetation, which has been left to protect the basin, has been destroyed by anthropogenic activities [12]. There are dry and rainy seasons in the study area. The mean annual rainfall is 1,220 mm. The average yearly temperature is 25.8°C [25]. Average humidity is high and ranges between 75 and 85%. The yearly evapotranspiration is 1500 mm. The average runoff is 2774 mm^3^ [27].

### 2.2. Study Design and Sampling Site Selection

Eighteen (18) fish samples from sampling sites S_8_ and S_9_ in the Asunafo South district of the Tano Basin were studied between November 2016 and October 2017. The levels of seven trace metals mercury (Hg), cadmium (Cd), lead (Pb), copper (Cu), arsenic (As), zinc (Zn), and chromium (Cr) in the muscles of fish samples from River Tano were studied. The selection of these metals was based on their toxicity, medical importance, bioaccumulation, and persistence [28]. The study area was mapped and geo-referenced with the aid of a hand-held Garmin 62SC Geographical Positioning System appliance [12].

### 2.3. Sampling and Sample Treatment

Eighteen (18) fish samples, which were made up of ten (10) species, were studied. The species were *Mormyrus rume, Leptocypris niloticus, Oreochromis niloticus, Chrysichthys johnelsi, Clarias gariepinus,* and *Parachanna obscura, Sarotherodon melanotheron, Ctenopoma gariepinus, Sarotherodon galilaeus and Ctenopoma kingsleyae*. Out of the 18 fish samples, 10 were males, while eight were females. Fish traps were set up at sites S_8_ and S_9_ and left to stay overnight. The fish caught in the net were collected and sorted according to species into brand-new polyethylene zip bags. They were zipped, labeled, and stored in an ice chest on ice. The length and sex of the fish samples were determined with the aid of officers from the fisheries Department of the Bono Region. The samples collected were transported to Ghana Atomic Energy Commission, where the analyses of heavy metals were done.

### 2.4. Analysis of Samples

The acid digestion method which was previously described by Benson et al. [29], Huang et al. [26], and Morshy et al. [9] was used. Each fish sample was washed three times with deionized water to avert any possible contamination [30, 31]. Each washed sample was dissected using stainless steel scalpels to remove the muscle. The muscle (which is the most edible part) grounded and homogenised using the domestic food blender as previously described by Rajeshkumar and Li [3]. One gram (1.0 g) of the powdered sample was digested using an automatic microwave system, a mixture of HNO_3_ : H_2_O_2_, deionized water in the ratio of 5 : 2:1 as described previously by Huang et al. [26]. The mixture was left at room temperature to cool, after which it was diluted with 20 mL of distilled water and filtered. The filtrate was kept and analysed for heavy metals (Cd, Cu, Pb, Zn, As, Hg, and Cr) using PerkinElmer (PinAACle 900T) Atomic Absorption Spectrophotometer (AAS). The analysis of Hg followed the hydride generation method of AAS where cold vapour was used. For quality control, the HNO_3_ and H_2_O_2_ used in this analysis were all guaranteed reagents. Duplicate samples were analysed and for each sample, trace metal concentration in mg/kg per fish sample was then calculated using

(1) $$\begin{array}{c} \text{concentration of the metal in mg }/\text{ kg of fish}=\frac{\text{AAS reading }\times \text{ volume of the extract}}{\text{mass of fish digested}}. \end{array}$$

### 2.5. Data Analysis

The data obtained were entered in EXCEL spreadsheet and imported into *R* software for analysis. The mean and standard deviation for each heavy metal was calculated [12]. Multivariate statistical approach including principal component analysis (PCA) was performed to determine the spread of metals in fish using JMP statistical software v. 10 (SAS Institute). The principal components were extracted with eigenvalues more than one through varimax rotation. The purpose of this analysis was to identify the possible source of heavy metals [32]. In this data analysis, *p* value < 0.05 was assumed to be statistically significant.

Fulton condition factor (*Q*) was used to assess the length–weight relationship of the fish samples and the fish conditions in the river according to (2), which was previously described and used by Ahmed et al. [33], Sekitar [34], and Jin et al. [35]:

(2) $$\begin{array}{c} Q=100\times \frac{w}{L^{3}}, \end{array}$$

where *W* is the total body weight of fish in grams (*g*), *L* is the total length of fish in centimeters (cm), and *Q* is the Fulton's condition factor: *Q* ≤ 1 means the condition is poor, *Q* = 1.2 means the condition is moderate, and *Q* ≥ 1.4 means the condition is proportionally good [34, 36].

Human health risk assessment was computed using USEPA [37] methodology with some modifications as previously described by Huang et al. [26]. The estimated daily intake (EDI) for each heavy metal in muscle of a given fish sample was calculated using the product per capita fish consumption in Ghana (25 kg/person/year), as given by MoFA [38]. The measured metal concentration (mg/kg) was divided by the average body weight according to

(3) $$\begin{array}{c} \text{EDI}=\frac{\text{FIR}\times C}{\text{BW}}, \end{array}$$

where *F*_IR_ is the ingestion rate of fish and fish products, which is 25 kg/person/year per adult [38], *C* is the measured metal concentration in fish in mg/kg, and BW is the average body weight = 70 kg for adults.

Target hazard quotient (THQ) was used to compute the potential noncarcinogenic risk assessment of metals in fish samples in this study. The THQ was expressed as the ratio of the EDI to an oral reference dose *RfD* in *μ*g/kg/day value as indicated by (4) and previously used by USEPA [37]. The reference doses (RfD) for the metals Cr, Zn, As, Cd, Pb, and Hg in *μ*g/kg/day are 3, 300, 0.3, 1.0, 1.5, and 0.3, respectively [26, 39].

(4) $$\begin{array}{c} \text{THQ}=\frac{\text{EDI}}{RfD}. \end{array}$$

A THQ >1 implies that the exposed populace will experience adverse health risks [18, 26]. Alternatively, a THQ <1 means noncarcinogenic risk for the exposed consumers. The carcinogenic risk index (CRI) was computed for As, Cd, and Cr in the fish samples using (5) as previously used by Huang et al. [26]. The oral intake of carcinogenic slope factor (SF) for As, Cr, and Cd is 0.38, 0.50, and 1.50, respectively [26, 33].

(5) $$\begin{array}{c} \text{CRI}=\frac{\text{FIR}}{\text{BW}}\times \text{SF}\times C, \end{array}$$

where CRI is a carcinogenic risk indicator, and CRI <1.0 × 10–6 which means that the fish is safe for human consumption. Alternatively, CRI >1.0 × 10–4 means excessive carcinogenic risks [26, 37].

## 3. Quality Control and Quality Assurance

Strict QC and QA protocols were observed in terms of precision, accuracy, and representativeness. All instruments used in this study were calibrated and validated using the specificity method as previously described by El-Gawad [40]. The glassware used in this study were soaked in 10% of HNO_3_ overnight and washed with deionized water several times and dried before using them. Acid digestion of the samples was validated by preparing, digesting, and analyzing nitric and distilled water in the same way as the fish samples. Deionized organic-free water samples were used as blanks. These were extracted and analysed in the same way as the real samples. During the digestion of fish samples, certified reference materials, SRM 8704 sourced from the National Institute of Standards and Technology, US, were included and prepared in the same way as the fish samples. The recovery ranged between 98% and 104%.

## 4. Results and Discussion

### 4.1. Fish Size, Weight, and Sex

The size, weight, and sex of fish samples are indicated in Table 1. The male fish samples were insignificantly longer and wider than the female fish samples. The mean length of male fish samples was 28.8 ± 14.4 cm, while that of the female was 19.7 ± 3.35 cm. The mean width of the male fish was 7.32 ± 2.33 cm, while that of the female was 5.76 ± 1.17 cm. The male fish samples were significantly heavier than the female fish samples (*p* < 0.05). The average weight of the male fish samples was 120.10 ± 4.0 g, while that of the female fish samples was 70.33 ± 0.9 g. The measured weight and size of the fish samples in this study are comparable to what was reported by Bawuro et al. [41] in fish samples from Lake Geriyo in Nigeria. Ahmed et al. [33] reported on fish samples in China, where the female fish samples were bigger and heavier than the male fish samples, contrary to what observed in this study. Variations in fish size, weight, and sex influence metabolic activity and contaminant levels [41].

### 4.2. Fulton Condition Factors of Fish Samples

Fulton condition factors were used to express the fish conditions in the river. The results are shown in Table 2. The results showed that the Fulton condition factor (*Q*) was in the range of 1.0–2.53. *Mormyrus rume* recorded the least *Q* value. The highest was recorded by *Oreochromis niloticus* to suggest that the condition of *Mormyrus rume* was poor, whereas that of *Oreochromis niloticus* was proportionally good. Fulton condition factor (*Q*) is used as an index to assess the health conditions of fish in the aquatic environment [35]. Fish with *Q* values greater than 1.4 are said to be in good condition, whereas those with *Q* values ≤ 1 are said to be in poor conditions [34]. Fish with *Q* values of 1.2 are said to be in moderate conditions [33]. In this study, *Mormyrus rume* recorded *Q* value of 1.0 to suggest poor conditions. *Sarotherodon galilaeus* fish recorded *Q* value of 1.12 to suggest a moderate condition. The rest recorded Q values greater than 1.4 to suggest proportionally good conditions. Poor fish conditions similar to what was recorded in *Mormyrus rume* were also recorded by Ahmed et al. [33] in the Karnaphuli River.

### 4.3. Heavy Metals in the Muscles of Fish Samples from River Tano

The results for the mean trace metals in the muscles of fish samples are presented in Table 3. The results showed that, apart from Cu, which was below the detection limits, the rest of the heavy metals studied were detected in the muscles of all fish samples. The nondetection of Cu in the muscles of fish samples may be due to lower concentrations in the river [10]. Cu levels ranging from 0.03 to 0.51 *μ*g/g were reported by Rajeshkumar and Li [3] in fish samples from Taihu Lake in China, contrary to what was observed in this study. Huang et al. [26] also reported 8.33 mg/kg Cu in fish samples from Northeast China.

The results of Pb levels analysed in fish samples were low. They range from <0.5 mg/kg fresh weight in *S. melanotheron* to 0.16 ± 0.05 mg/kg in *O. niloticus* (Table 3). A decreasing order of Pb accumulation in the muscles of fish species studied is shown in Table 4. From the results, the highest concentration of Pb in fish muscles was in *O. niloticus* (Table 4). Similarly, Cd concentrations recorded in the fish samples were also low ranging from <0.3 mg/kg in the fresh body weight in *O. niloticus, S. galilaeus and C. kingsleyae* to 0.03 ± 0.02 mg/kg in *M. rume* (Table 3). An order of Cd levels in the muscles of the fish species studied is shown in Table 4. The results show that a slightly elevated level is established in the muscles of *M. rume.* The levels of As in the fish samples analysed were high (Table 3). They ranged from 1.01 ± 0.08 mg/kg in *C. gariepinus* to 3.00 ± 0.01 mg/kg of fresh weight of *M. rume*. The order of As accumulation in the muscles of the fish species studied can be established from Table 4, with the highest As accumulation recorded in *M. rume.* The results showed elevated levels of Hg in all fish samples (Table 3). The results showed that the levels of Hg in the fish samples ranged from 0.58 ± 0.69 mg/kg of fresh body weight of *O. niloticus* to 2.52 ± 0.70 mg/kg in *C. kingsleyae.* The order of Hg accumulation in the muscles of the fish species studied recorded the highest Hg concentration in *C. kingsleyae* (Table 4). The levels of Cr concentrations in the fish samples studied are high (Table 3). They ranged from 16.10 ± 0.2 mg/kg in *M. rume.*to 57.9 ± 4.2 mg/kg in *Sarotherodon galilaeus*. The order of Cr accumulation studied in the muscles of the fish species showed the highest accumulation in *S. galilaeus* (Table 4). Zn levels in the fish samples were low (Table 3). They ranged from 8.42 ± 6.15 mg/kg fresh weight in *S. melanotheron* to 12.5 ± 3.5 mg/kg in *S. galilaeus* (Table 3). A decreasing order of Zn accumulation in the muscles of fish species studied is shown in Table 4. From the results, the highest concentration of Zn in fish muscles was in *S. galilaues* (Table 4).

The general order of increasing metal accumulation in the muscles of fish species is Cu < Cd < Pb < Hg < As < Zn < Cr. The least metal found in the muscles of the fish species studied was Cu and the highest was Cr (Table 4). The observed trend in the low accumulation of Cu and Cd may be due to their low tendency to accumulate in the muscles but high affinity to metabolic organs such as liver and kidney [42]. Pb highly accumulates in the bones compared with the muscles, and this may account for low the accumulation in the fish muscles. Higher accumulation of Zn in the muscle may be attributed to its being important for the regular physiological and central functions of fish [17]. The observed levels of Cu, As, Pb, Cr, and Cd in the fish muscles studied were similar to the observations made by Ahmed et al. [33] in fish samples from Karnaphuli River in Bangladesh. Huang et al. [26] also reported on the levels of Hg, As, Pb, Cu, Zn, Cr, and Cd in fish samples from surface water bodies in Northeast China, which were lower than the findings in this study. Awuah [31] reported on As levels in fish samples from the downstream of River Tano and River Ankobra in Ghana, which were comparable to the findings in this study. Asare-Donkor and Adimado [1] reported on Hg levels in fish similar to this study from the downstream of River Tano. Higher levels of Cr in fish samples from Douglas Creek in the Qua Iboe Estuary, which were higher than the findings in this study, were reported by Benson et al. [29]. In China, higher Cr concentrations similar to the findings in this study were reported by Rejeshkumar and Li [3] in the Meiliang Bay and Taihu Lake. Heavy metals in the muscles of fish may be attributed to agricultural and municipal runoff [1, 3, 10, 29].

The limits for heavy metal in fish muscles have been set to safeguard public health. The limits differ from country to country and organization to organization. In this study, comparisons were made between the measured heavy metals in the fish muscle studied and the Food and Agriculture Organization limits [43, 44] and FAO/WHO [45]. The results showed that Cd, Pb, and Zn were lower than the respective limits of FAO/WHO in fish muscles (Table 3). However, thre recorded levels of As and Hg exceeded the FAO/WHO limits in fish muscles, which are 0.5 mg/kg for each of them. The FAO/WHO permissible limits of Cd, Pb, and Zn in fish muscles are, respectively, 0.05 mg/kg, 0.2 mg/kg, and 30 mg/kg. The United States Food and Drug Administration [46] recommends 12–13 mg/kg as the limit for Cr in fish muscles. Comparison with the levels of Cr in the muscles of fish samples studied revealed that Cr levels in fish exceeded the limits in fish muscles (Table 3). Ingestion of fish polluted with high levels of Hg, As, and Cr is associated with health problems, including kidney, liver, and skin cancers [1, 12, 26]. For example, the Minamata disease in Japan in the 60s was attributed to methylmercury (MeHg) exposure through seafood consumption [28].

### 4.4. Risk Assessment of Heavy Metals in the Muscles of Fish Species

#### 4.4.1. Noncarcinogenic Risk Analysis of Metals in Fish Samples from River Tano

The results for the noncarcinogenic risk assessments for the adult groups are presented in Table 5. The results showed that the target hazard quotients (THQs) for Cd, Zn, and Pb in fish samples were less than one for adults to suggest no possible noncarcinogenic risks in the consumption of all the fish species studied. However, THQ values for As, Cr, and Hg in all the fish species studied for the adult's groups were greater than 1 to suggest possible noncarcinogenic risks to consumers of all the fish species from the river studied. THQ >1 for Hg observed in this study has also been reported by Asare-Donkor and Adimado [1] in fish samples from the downstream of River Tano. In Youngshu Island in China, Wu et al. [47] reported on THQ >1 for Cr in fish samples, comparable to the findings in this study. Contrary to the findings in this study, Huang et al. [26] computed THQ <1 for Cr, As, and Hg in fish samples from Northeast China. In the same study, however, THQ <1 was recorded for Pb and Cd, which was similar to the findings in this study. Mohammadi et al. [48] reported on THQ <1 for Cd, Pb, and Zn, which was comparable to the findings in this study for fish samples from Khorramabad in Iran. Benson et al. [29] reported THQ <1 for Zn, Pb, and Cd in fish samples from Douglas Creek in the Qua Iboe Estuary, which was comparable to the findings in this study. Ashraf et al. [19] also recorded high THQ in some fish samples from Peninsular Malaysia, which is comparable to the computed THQ values in this study. The high THQ values from this study suggest that consumption of fish from River Tano has adverse effects [1, 29].

### 4.5. Carcinogenic Risk Assessment of Metals in Fish Samples

The carcinogenic risk assessment results for Cd, As, and Cr in fish samples are presented in Table 6. The results showed that the calculated cancer risk index (CRI) for each metal was higher than 1.0 × 10−4 in all fish samples for the adult groups of people. A given fish sample is said to be safe for consumption when the CRI value is less than 1.0 × 10−6 for a given metal [26]. The observed CRI values in this study suggest potential carcinogenic risks through the consumption of fish samples from River Tano [26, 33, 39].

### 4.6. Principal Component Analysis (PCA)

**Figure 2:**
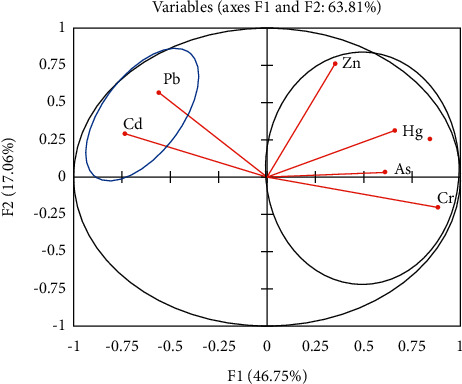
Principal composite analysis for heavy metals in fish samples.

The results for the PCA are shown in Table 7 and Figure 2. The results indicated that two (2) components with eigen factors greater than 1, which accounted for 63.81%, were extracted. The first component (F1) accounted for 46.7% of the loading and was dominated by Hg, As, Zn, and Cr. The second component (F2), which accounted for 17.06%, was dominated by high loadings of Pb and Cd. The observed connotation between Cd and Pb in this study is similar to what was observed by Wang et al. [49] in the Huaihe River in China and was attributed to applying agrochemicals and industrial discharge. Similarly, the association between Hg, As, Zn, and Cr may suggest that they are also coming from a common source, possibly an agricultural runoff, pesticide application, or geological sources [10]. In the Weija Reservoir in Ghana, Ansah et al. [50] reported on a strong association between As and Hg similar to what was observed in this study.

## 5. Conclusion

The levels of trace metals in fish samples from River Tano and the risks associated with their consumption have revealed that the levels of Cu in the muscles of all fish samples studied were below detection limits. However, some levels of As, Cd, Hg, Pb, Zn, and Cr were detected in muscles of all fish samples. *Mormyrus rume* fish species was under stress in the river, whereas the remaining fish species were proportionally in good condition. The measured levels of Cd, Zn, Cu, and Pb were within the Food and Agriculture Organization limits of metals in fish muscles. However, the levels of Hg, As, and Cr in all fish samples exceeded the respective recommended permissible levels in fish. The target hazard quotients for Cd, Zn, and Pb in fish samples were less than 1, suggesting no possible noncarcinogenic risk of metals for adults. On the other hand, As, Cr, and Hg recorded target hazard quotient values greater than 1 for adults in all fish sample, suggesting possible noncarcinogenic risks to consumers of fish from the river. Additionally, high carcinogenic risks were recorded for As, Cr, and Hg for all fish samples in adults, making the consumption of fish from River Tano unsafe. Strong association was found between Hg, As, Zn, and Cr and between Pb and Cd suggesting a common source, possibly industrial effluent discharge and agricultural runoff. Continuous monitoring of trace metals in fish from River Tano and the enforcement of the buffer zone policy in the Tano Basin are highly recommended.

## Figures and Tables

**Table 1:** Comparison between female and male fish dimensions.

Dependent variables	Sex	*n*	Weight (*g*) ± SD	Mean (cm) ± SD	*t* value	*P* value
Length	Male	10	N/A	28.8 ± 14.4	1.74	0.102
	Female	8	N/A	19.7 ± 3.35		
Width	Male	10	N/A	7.32 ± 2.33	1.72	0.105
	Female	8	N/A	5.76 ± 1.17		
Weight	Male	10	120.10 ± 4.3	14.76 ± 4.56	1.70	0.012^a^
	Female	8	70.33 ± 0.9	10.32 ± 1.45		

^a^Difference is significant at *p* < 0.05.

**Table 2:** Weight-length relationship and condition factor.

Fish species	Weight (*g*) ± SD	Length (cm) ± SD	*Q* value	Fish condition
*Mormyrus rume*	531 ± 0.19	37.5 ± 0.01	1.0	Poor
*Leptocypris niloticus*	308 ± 0.54	26.5 ± 0.00	2.19	Proportionally good
*Oreochromis niloticus*	252 ± 0.31	21.5 ± 0.00	2.53	Proportionally good
*Chrysichthys johnelsi*	266 ± 0.75	22.0 ± 0.02	2.50	Proportionally good
*Clarias gariepinus*	108 ± 0.38	17.0 ± 0.01	2.20	Proportionally good
*Parachanna obscura*	99.5 ± 0.21	19.0 ± 0.05	1.45	Proportionally good
*Saro. melanotheron*	58.2 ± 1.01	16.0 ± 0.02	1.42	Proportionally good
Ctenopoma *gariepinus*	91.0 ± 0.54	17.0 ± 0.00	1.85	Proportionally good
*Sarotherodon galilaeus*	73.5 ± 0.59	18.7 ± 0.01	1.12	Moderate
*Ctenopoma kingsleyae*	120 ± 0.20	20.4 ± 0.06	1.41	Proportionally good

The *Q* value means the Fulton condition factor.

**Table 3:** Levels of heavy metals in the muscles of fish samples from River Tano.

Fish species	Heavy metal concentration ± SD (mg/kg)						
	Cu	Pb	Cd	As	Hg	Cr	Zn
*M. rume*	<0.3	0.12 ± 0.06	0.03 ± 0.02	3.00 ± 0.01	1.01 ± 0.03	14.9 ± 0.02	9.91 ± 1.30
*L. niloticus*	<0.3	0.10 ± 0.01	0.01 ± 0.05	1.55 ± 0.10	1.1 ± 0.14	32.2 ± 14.3	11.7 ± 4.67
*O. niloticus*	<0.3	0.16 ± 0.05	<0.3	1.52 ± 0.70	0.58 ± 0.69	27.0 ± 36.6	10.8 ± 2.10
*C. johnelsi*	<0.3	0.08 ± 0.04	0.02 ± 0.10	2.17 ± 0.20	1.57 ± 2.0	33.0 ± 15.5	12.2 ± 8.01
*C. gariepinus*	<0.3	0.13 ± 0.03	0.01 ± 0.07	1.01 ± 0.08	1.00 ± 0.04	16.1 ± 2.40	10.8 ± 2.76
*P. obscura*	<0.3	0.04 ± 0.01	0.01 ± 0.01	1.57 ± 0.65	1.58 ± 0.74	17.9 ± 11.3	12.4 ± 3.38
*S. melanotheron*	<0.3	<0.5	0.01 ± 0.01	1.09 ± 0.17	2.07 ± 1.90	17.8 ± 0.07	8.42 ± 6.15
*C. gariepinus*	<0.3	0.01 ± 0.10	0.01 ± 0.06	2.61 ± 0.55	2.01 ± 1.40	40.4 ± 10.4	12.5 ± 2.17
*S. galilaeus*	<0.3	0.02 ± 0.01	<0.3	2.57 ± 0.82	2.02 ± 1.39	57.9 ± 4.2	12.5 ± 3.5
*C. kingsleyae*	<0.3	0.02 ± 0.04	<0.3	2.54 ± 0.69	2.52 ± 0.70	52.4 ± 20.4	10.1 ± 12.7

**Table 4:** Heavy metals' accumulation in the muscles of fish species.

Metal	Order of metal accumulation in fish muscles
Pb	*S. melanotheron* < *C*. *gariepinus* < *S*. *galilaeus* < *C*. *kingsleyae* < *P*. *obscura* < *C*. *gariepinus* < *L*. *niloticus* < *M*. *rume* < *C*. *gariepinus* < *O*. *niloticus*
Cd	*O. niloticus* < *C*. *gariepinus* < *O*. *niloticus* < *C*. *kingsleyae* < *S*. *melanotheron* < *P*. *obscura* < *C*. *gariepinus* < *L*. *niloticus* < *C*. *johnelsi* < *M*. *rume*
As	*C. gariepinus* *<* *S. melanotheron* *<* *O. niloticus* *<* *L. niloticus* *<* *P. obscura* *<* *C. johnelsi* *<* *C. kingsleyae* *<* *L. niloticus* *<* *S. galilaeus* *<* *M. rume*
Hg	*O. niloticus* *<* *C. gariepinus* *<* *M. rume* *<* *L. niloticus* *<* *C. johnelsi* *<* *P. obscura* *<* *C. gariepinus* *<* *S. galilaeus* *<* *S. melanotheron* *<* *C. kingsleyae*
Cr	*M. rume* *<* *C. gariepinus* *<* *S. melanotheron* *<* *P. obscura* *<* *C. johnelsi* *<* *L. niloticus* *<* *C. johnelsi* *<* *C. gariepinus* *<* *C. kingsleyae* *<* *S. galilaeus*
Zn	*S. melanotheron* *<* *M. rume* *<* *C. kingsleyae* *<* *O. niloticus* *<* *C. gariepinus* *<* *L. niloticus* *<* *P. obscura* *<* *C. johnelsi* *<* *C. gariepinus* *<* *S. galilaeus*

**Table 5:** Noncarcinogenic analysis of metals in fish samples.

Fish species	Pb		Cd		As		Hg		Cr		Zn	
	EDI	THQ	EDI	THQ	EDI	THQ	EDI	THQ	EDI	THQ	EDI	THQ
*Mormyrus rume*	0.04	0.03	0.01	0.01	1.07	3.6	0.36	1.2	5.32	1.8	3.5	0.01
*Leptocypris niloticus*	0.38	0.25	0.01	0.01	0.55	1.8	0.39	1.3	11.5	3.8	4.18	0.01
*Oreochromis niloticus*	0.08	0.38	N/A	N/A	0.05	1.8	0.21	0.69	9.64	3.2	3.85	0.01
*Chrysichthys johnelsi*	0.03	0.19	0.01	0.01	0.77	2.6	0.56	1.9	11.8	3.9	4.36	0.01
*Clarias gariepinus*	0.05	0.30	0.01	0.01	0.36	1.2	0.38	1.2	5.75	1.9	3.85	0.01
*Parachanna obscura*	0.014	0.09	0.004	0.003	0.56	1.9	0.56	1.9	6.39	2.1	4.4	0.15
*Sarotherodon melanotheron*	N/A	N/A	0.004	0.004	0.39	1.3	0.73	2.5	6.35	2.1	3.01	0.01
*Ctenopoma gariepinus*	0.004	0.002	0.004	0.004	0.93	3.1	0.72	2.4	14.4	4.8	4.46	0.01
*Sarotherodon galilaeus*	0.007	0.005	N/A	N/A	0.917	3.0	0.721	2.4	20.8	6.9	4.46	0.01
*Ctenopoma kingsleyae*	0.007	0.005	N/A	N/A	0.906	3.0	0.899	3.0	18.7	6.2	3.605	0.01

N/A means not applicable.

**Table 6:** Carcinogenic risk index for As, Cr, and Cd metals in fish samples.

Fish species	Cd		As		Cr	
	EDI	CRI	EDI	CRI	EDI	CRI
*Mormyrus rume*	0.01	1.5 .510^−2^	1.07	4.0 × 10^−1^	5.32	2.66
*Leptocypris niloticus*	0.01	1.5 .510^−2^	0.55	2.1 × 10^−1^	11.5	5.75
*Oreochromis niloticus*	N/A	N/A	0.05	1.9 × 10^−2^	9.64	4.82
*Chrysichthys johnelsi*	0.01	1.5 .510^−2^	0.77	2.9 .910^−1^	11.8	5.9
*Clarias gariepinus*	0.01	1.5 .510^−2^	0.36	1.4 .410^−1^	5.75	2.9
*Parachanna obscura*	0.004	6.0 .010^−3^	0.56	2.1 .110^−1^	6.39	3.2
*Sarotherodon melanotheron*	0.004	6.0 .010^−3^	0.39	1.5 .510^−1^	6.35	3.2
*Ctenopoma gariepinus*	0.004	6.0 .010^−3^	0.93	3.5 .510^−1^	14.4	7.1
*Sarotherodon galilaeus*	N/A	N/A	0.917	3.5 .510^−1^	20.8	10.4
*Ctenopoma kingsleyae*	N/A	N/A	0.906	3.4 .410^−1^	18.7	9.35

N/A means not applicable.

**Table 7:** Varimax component matrix for heavy metals in fish.

	F1	F2	F3	F4	F5	F6	F7
Eigenvalue	3.2723	1.1945	0.9492	0.6333	0.5162	0.2501	0.1843
Variability (%)	46.7472	17.0645	13.5605	9.0471	7.3743	3.5734	2.6329
Cumulative (%)	46.7472	63.8117	77.3723	86.4194	93.7937	97.3671	100.000

## Data Availability

All data used in this manuscript are available at the discretion of the authors.
